# Thermal Radiation Shielding and Mechanical Strengthening of Mullite Fiber/SiC Nanowire Aerogels Using In Situ Synthesized SiC Nanowires

**DOI:** 10.3390/ma15103522

**Published:** 2022-05-13

**Authors:** Hui Xu, Xiaolei Li, Zongwei Tong, Baojie Zhang, Huiming Ji

**Affiliations:** Key Laboratory for Advanced Ceramics and Machining Technology of Ministry of Education, School of Materials Science and Engineering, Tianjin University, Tianjin 300072, China; xhvenus@163.com (H.X.); tongzongwei@tju.edu.cn (Z.T.); zhangbaojie@tju.edu.cn (B.Z.); jihuiming@tju.edu.cn (H.J.)

**Keywords:** SiC nanowires, mullite fiber preform, high compressive strength, infrared thermal shielding

## Abstract

Traditional solid nanoparticle aerogels have been unable to meet the requirements of practical application due to their inherent brittleness and poor infrared shielding performance. Herein, combining vacuum impregnation and high-temperature pyrolysis, a novel micro/nano-composite fibrous aerogel was prepared via in situ synthesis of silicon carbide nanowires (SiC NWS) in mullite fiber (MF) preform. During this process, uniformly distributed SiC NWS in the MF preform serve as an enhancement phase and also act as an infrared shielding agent to reduce radiation heat transfer, which can significantly improve the mechanical properties of the mullite fiber/silicon carbide nanowire composite aerogels (MF/SiC NWS). The fabricated MF/SiC NWS exhibited excellent thermal stability (1400 °C), high compressive strength (~0.47 MPa), and outstanding infrared shielding performance (infrared transmittance reduced by ~70%). These superior properties make them appealing for their potential in practical application as high-temperature thermal insulators.

## 1. Introduction

In aerospace and thermal power fields, high-efficiency thermal insulation materials with excellent mechanical, thermal, and chemical stability are urgently required to work at long-term high temperatures [1,2,3]. Ceramic nanoparticles aerogel is recognized as promising thermal insulation material with a series of superior performance-related features, such as light weight, ultrahigh porosity, large specific surface area, ultralow thermal conductivity at room temperature, etc. [3,4,5,6,7,8]. Despite these merits, the practical application of traditional ceramic nanoparticles aerogel is impeded by its inherent brittleness, poor high-temperature structural stability, and high radiation heat transfer [9,10,11,12]. Fiber-reinforced aerogels, with fibers that act as an effective skeleton, are beneficial to declining the volume shrinkage of aerogel at high temperatures by confining the aerogel to the internal pore structure formed by the adjacent fibers. With this strategy, the mechanical properties of aerogels are greatly enhanced, together with the slightly improved thermal stability [13]. However, fibers are easily debonded from the aerogel, owing to the poor adhesion between the aerogel and fiber surface [14,15]. In addition, the fabrication process of these materials generally involves solvent exchange, gel modification, and supercritical drying, which leads to a tedious preparation cycle and high cost [16,17,18]. Furthermore, some new ceramic aerogels with hyper-elasticity assembled from flexible ceramic nanostructures are reported widely, including oxide nanofiber aerogels (e.g., SiO_2_ [19], ZrO_2_ [20]), SiC nanowires aerogels [21,22], Si_3_N_4_ nanobelt aerogel [23,24], BN foam and aerogel [25,26,27], etc. These ceramic aerogels possess high reversible compressibility and improved thermal stability, but current thermal insulation materials are still dominated by hard materials. Table 1 summarizes relevant studies in the literature on different types of aerogels mentioned above and makes a detailed comparison of the properties of various aerogels.

In recent years, a new kind of ceramic aerogel with fibers as matrix and nanostructured materials as reinforcement is being developed [12,28,29,30]. Micro and nano fibers interlace together to form porous structure similar to traditional aerogels, but overcome the brittleness of traditional aerogel, which exhibits higher heat resistance and compressive strength. Among them, mullite-fiber-based aerogels have gained great interest owing to their excellent comprehensive properties (e.g., excellent heat and fire resistance, outstanding thermal shock resistance, and low thermal conductivity at room temperature) [31,32,33]. On the basis of this, many mullite-fiber-based aerogels have been reported. For instance, Zhao and Yi et al. [12,28,29] in situ synthesized mullite whiskers in the porous mullite fiber ceramic. This micro/nano-ceramic fiber aerogel with a secondary-pore structure exhibited good mechanical performance and low thermal conductivity at room temperature.

However, in high-temperature environments, the heat transfer of materials is mainly accomplished via thermal radiation. According to Planck’s law, the higher the temperature, the greater the proportion of thermal radiation in heat transfer. The wavelength of thermal radiation applied to insulation materials is mainly concentrated at 1–8 μm [34], but the infrared transmittance of fiber aerogels is high in this band [30,35]. At present, decreasing infrared transmittance of fiber aerogels is mainly through the construction of high reflectivity coating on the fiber surface (TiO_2_ coating [36], SiC coating [35], etc.) and electrospinning micro/nano-ceramic fibers with internal composite infrared shielding phase [37,38,39]. Xu et al. [35] prepared SiC coatings on the surface of mullite fibers, which increased the infrared extinction coefficient of the fibers and reduced the thermal conductivity of the composites by 20% at 1100 °C. However, in high-temperature environments, the coating is easily detached from the fiber surface. Moreover, electrospinning technology is inefficient, which is unfavorable for practical high-temperature applications. Therefore, this issue highlights the need to prepare aerogel materials with excellent structural stability and infrared thermal shielding performance.

In the range of 1–8 μm, SiC is capable of acting as an excellent infrared shielding material to improve the high-temperature thermal insulation of mullite fiber aerogel owing to its high infrared reflection and absorption capacity [35,40]. Similarly, one-dimensional SiC nanostructures have been shown to have a high infrared reflection and absorption capacity and exhibited an infrared thermal radiation shielding effect [41,42]. In addition, in situ synthesis of nanowires and whiskers in ceramic fibrous insulation materials with high porosity has been widely used owing to its characteristics of flexibility and low thermal conductivity [12,30,43]. It is expected to overcome the brittleness of traditional ceramic insulation fibers, and prepare high effective thermal insulation materials with excellent mechanical properties. A large number of methods for preparing SiC nanowires have been reported [44,45,46,47,48,49]. Among these methods, carbon and silicon sources can achieve uniform mixing at molecular and atomic scales in a short time by the sol–gel process [45,49], so as to synthesize nanowires with high yield at relatively low temperatures. Additionally, the template method regulates the growth of SiC NWS through a three-dimensional framework, which contributes to the uniform distribution of SiC NWS [49].

In this study, a mullite fiber/silicon carbide nanowire composite aerogel (MF/SiC NWS) was prepared using in situ synthesized silicon carbide nanowires (SiC NWS) in a mullite fiber (MF) preform, applying a combination of sol–gel, vacuum impregnation, and high-temperature pyrolysis techniques. The microstructure, mechanical, and thermal properties of MF/SiC NWS were studied. Moreover, the growth mechanisms of SiC NWS and heat insulation mechanisms of MF/SiC NWS were analyzed.

## 2. Materials and Methods

### 2.1. Preparation of MF Preform

The first step was to prepare MF preform. Mullite fiber s were prepared from mullite fiber cotton (99.5%, Zhejiang Hongda Crystal Fiber Co., Ltd., Zhejiang, China) by shearing and slag removal with a high-speed blender. Boracic acid (H_3_BO_3_, AR grade, Tianjin Guangfu Fine Chemical Research Institute Co., Ltd., Tianjin, China) and silica sol (Jinan Yinfeng Silicon Products Co., Ltd., Shandong, China) were selected as high-temperature binders. Soluble starch (C_12_H_22_O_11_, AR grade, Shanghai Macklin Biochemical Co., Ltd., Shanghai, China) was selected as the low-temperature binder. Firstly, as-prepared mullite fiber, soluble starch, H_3_BO_3_, and silica sol were mixed in 50 mL deionized water, with a weight ratio of 0.3:1:9:10, by magnetic stirring for 20 min, yielding a uniform fiber/binder mixture slurry. Then, the obtained mixture slurry was prepared into a wet billet using vacuum filtration and molding. Finally, the wet billet was dried at 90 °C for 4 h, followed by sintering at 1200 °C for 2 h, with a heating rate of 5 °C·min^−1^ in the air, further attaining MF preform. The bulk density of MF preform is 0.20 ± 0.02 g·cm^−3^, with a diameter and height of 40 mm and 10 mm, respectively.

### 2.2. Preparation of MF/SiC NWS

MF/SiC NWS were fabricated via vacuum impregnation and high-temperature pyrolysis with TEOS as silicon source and glucose as carbon source. In a typical process, TEOS (AR grade, Shanghai Macklin Biochemical Co., Ltd., Shanghai, China), deionized water, and EtOH with a molar ratio of 1:16:4 were mixed by stirring for 2 h to obtain a homogenous solution. Then, HNO_3_ was added into the as-prepared solution to adjust the corresponding pH in the range of 3–4 to promote SiO_2_ hydrolysis, thus obtaining SiO_2_ precursor sol. Meanwhile, glucose solution was prepared by dissolving glucose (C_6_H_12_O_6_▪H_2_O, AR, Tianjin Yuanli Chemical Co., Ltd., Tianjin, China) in deionized water, followed by a water bath at 40 °C for 0.5 h. Subsequently, glucose solution and SiO_2_ precursor sol were mixed with the molar ratio of C: Si = 4:1, by magnetic stirring for 0.5 h, yielding precursor sol with a SiO_2_ concentration of 0.6 mol L^−1^. For direct comparison, precursor sols with SiO_2_ concentrations of 0.4, 0.6, 0.8, and 1.0 mol L^−1^ were also prepared by varying the TEOS content in the solution. After that, MF preform was entirely immersed into the as-prepared precursor sol under vacuum at 50 °C for 2 h and then dried at 60 °C to obtain MF/dry gel. Finally, MF/dry gel was pyrolyzed at 1400 °C for 2, 3, and 4 h in the airtight alumina crucible in Ar to prepare MF/SiC NWS. The bulk density of MF/SiC NWS is 0.22 ± 0.02 g·cm^−3^, with a diameter and height of 40 mm and 10 mm, respectively.

### 2.3. Characterization

Bulk density was calculated using ρ = m/V (where ρ, m, and V are bulk density, mass, and volume of the MF preform and MF/SiC NWS, respectively). Pore-size distribution was analyzed using mercury intrusion porosimetry (Auto Pore V 9600). Morphologies of the samples were characterized via field-emission scanning electron microscopy (FESEM, S4800, JEOL, Akishima, Japan). The crystalline structure and phase compositions of SiC NWS were examined via field-emission transmission electron microscopy (TEM, JEM2100-F, JEOL, Akishima, Japan) with an energy-dispersive spectrometer (EDS). A thermal analyzer (Netzsch Sta 449C, GER) was used to record the curves of thermogravimetry (TG) of MF/SiC NWS from room temperature to 1500 °C in the air at a heating rate of 10 °C·min^−1^. Compression measurements of MF preform and MF/SiC NWS were measured using a universal testing machine with a loading speed of 0.05 mm·min^−1^ (XWW, Beijing Shengxin Detecting Instrument, Beijing, China). FTIR (Nicolet Avatar 360) and a UV–Vis–NIR spectrophotometer (Lambda 750, USA) were used to determine the infrared transmittance of the samples. The Thermograph of the MF/SiC NWS was recorded by an infrared thermal imager (Fluke TiX640 Expert HD, USA).

## 3. Results

### 3.1. Material Design and Morphology Analysis

**Figure 1:**
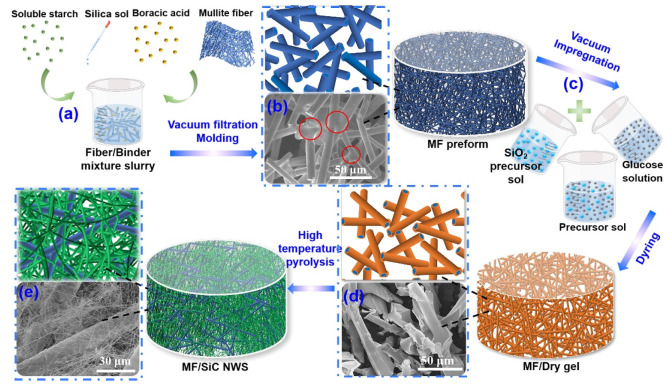
Schematic illustration of the synthesis process of (**a**,**b**) mullite fiber preform (MF), (**c**,**d**) MF/Dry gel, and (**e**) mullite fiber/silicon carbide nanowire composite aerogels (MF/SiC NWS).

The fabrication of MF/SiC NWS is schematically illustrated in Figure 1. In the preparation process of MF preform (Figure 1a,b), high-temperature binder adhered to the adjacent joints formed by overlapped mullite fibers to build a high-strength skeleton at high temperature. The fibers-lap pores were about 50–100 μm, providing enough space for the subsequent in situ synthesis of SiC NWS (Figure 1b). Moreover, for the preparation of precursor sol, the good water solubility of both glucose and SiO_2_ sol is beneficial for obtaining uniformly mixed sol (Figure 1c). Adding glucose also increased the viscosity of the precursor sol and facilitated the attachment of the precursor sol on the surface of mullite fibers. After vacuum impregnation, the precursor sol evenly covered the surface of mullite fibers, and the interior of the MF/dry gel remained a porous structure (Figure 1d). After the in situ synthesis of SiC NWS, the anisotropic nanowires were homogeneously distributed in the space among the MF preform and interweaved to form a secondary-pore structure (Figure 1e). Mullite fiber and silicon carbide nanowires were interlaced together to form a large number of micron and nanometer pores, which is similar to the porous structure of traditional aerogels.

There was no occurrence of spherical particles on the top of SiC NWS (Figure 1e), indicating the generation of SiC NWS is based on chemical vapor transport according to the vapor–solid reaction mechanism [21,46,47,48]. Therefore, the growth of SiC NWS can be effectively controlled by adjusting the gas-phase partial pressure of SiO and CO upon adjusting the precursor concentration and pyrolysis time, etc.

**Figure 2:**
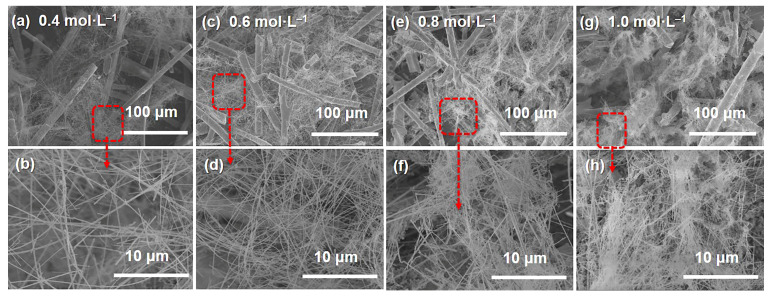
SEM images of MF/SiC NWS under different precursor sol concentration: (**a**,**b**) 0.4 mol·L^−1^; (**c**,**d**) 0.6 mol·L^−1^; (**e**,**f**) 0.8 mol·L^−1^; (**g**,**h**) 1.0 mol·L^−1^.

Figure 2 shows the effect of precursor concentrations on the microstructure of MF/SiC NWS pyrolyzed at 1400 °C for 4 h. As shown in Figure 2, the number of SiC NWS gradually increased with the rise in precursor concentration. When precursor concentration was 0.4 mol·L^−1^, the supply of raw materials in the system was insufficient. Therefore, it is difficult to guarantee continuous nucleation sites in the growth process of SiC NWS, resulting in a sparse distribution of SiC NWS (Figure 2a,b). While the precursor concentration increased to 0.6 mol·L^−1^, SiC NWS evenly distributed and became interwoven with each other in the MF preform, forming secondary pores on the basis of micrometer-sized pores interwoven with mullite fibers (Figure 2c,d). However, as the precursor concentration increased to 0.8 and 1.0 mol L^−1^, the content of SiC NWS increased noticeably (Figure 2f,h), but almost all of them finally gathered near the mullite fibers (Figure 2e,g), which may be caused by the limited interconnection of internal pore structure in MF preform after impregnated precursor sol, which limited the growth of SiC NWS is limited [49,50].

**Figure 3:**
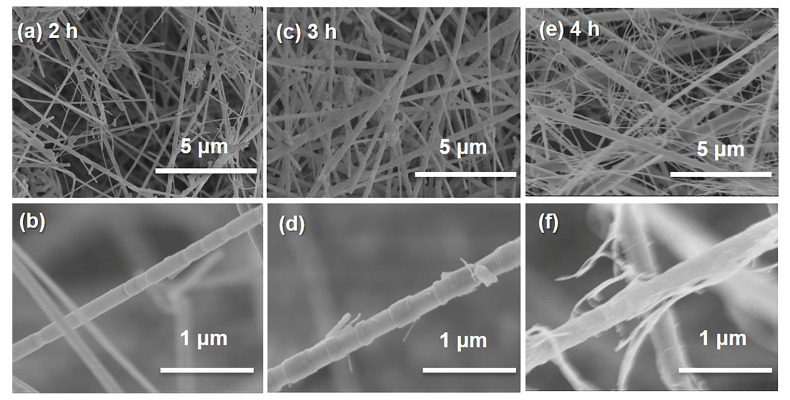
SEM images of MF/SiC NWS: the pyrolysis time: (**a**,**b**) 2 h; (**c**,**d**) 3 h; (**e**,**f**) 4 h.

Figure 3 shows the effect of pyrolysis time on the growth of SiC NWS at 1400 °C with a precursor concentration of 0.6 mol·L^−1^. When the pyrolysis time was 2 h, the overall distribution of SiC NWS was sparse, as shown in Figure 3a; the pore with around 2 μm diameter formed by the interweaving of nanowires, and the diameter of SiC NWS were about 100 nm (Figure 3b). Extending the pyrolysis time to 3 h, the diameter of prepared SiC NWS increased obviously and was around 200 nm. Additionally, the interlaced nanowires’ pore size decreased to about 1 μm (Figure 3c,d). Due to the lack of newly generated reactant gas in the stage of 3–4 h, the further growth of SiC NWS was significantly reduced. Newly generated SiC NWS were finer (~20 nm) than before (~200 nm) and connected with each other to form smaller size pores (~500 nm) (Figure 3e,f).

**Figure 4:**
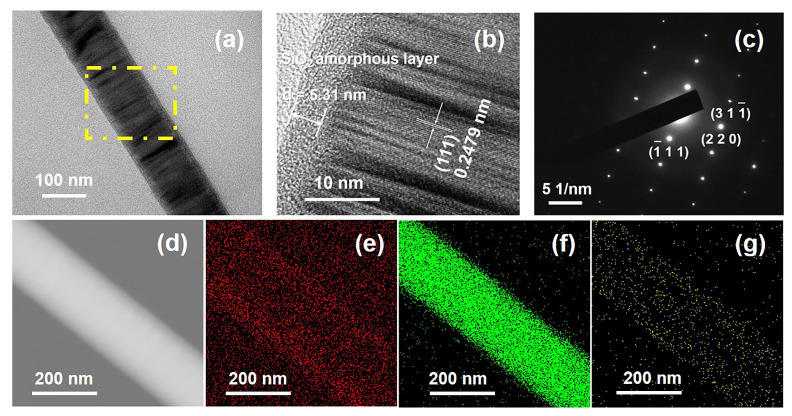
(**a**) TEM images of SiC NWS; (**b**) HRTEM image of SiC NWS; (**c**) SAED patterns of SiC NWS; (**d**–**g**) TEM image of SiC NWS and the corresponding EDX elemental mappings of C, Si, O elements.

As shown in Figure 4, the microstructure of the SiC NWS prepared at 0.6 mol/L and pyrolyzed at 4 h was further investigated via TEM, SEAD, and EDS techniques. The diameters of SiC NWS were in the range of 100−200 nm, and it contained an amorphous silica layer with a thickness of ~5.31 nm (Figure 4a,b). HRTEM image (Figure 4b) and the SAED pattern (Figure 4c) revealed that SiC NWS exhibited a perfect single crystal structure. The lattice fringe of 0.2479 nm was well indexed with the (111) planes of β-SiC (JCPDS card No. 29). The results of elemental mapping images indicated that the SiC NWS was mainly composed of C and Si, as well as lower O content (Figure 3d–g). The presence of O was related to the amorphous silica layer formed on the surface of SiC NWS.

Based on the above analysis, the sample with a precursor concentration of 0.6 mol L^−1^ and pyrolysis at 1400 °C for 4 h exhibited an ideal microstructure. SiC NWS with diameters of several tens or hundreds of nanometers were uniformly distributed in the MF preform and interconnected with mullite fibers to form a hierarchical pore structure, which is associated with excellent mechanical and thermal insulation performance and is discussed later.

### 3.2. The In Situ Formation Mechanism of SiC NWS

**Figure 5:**
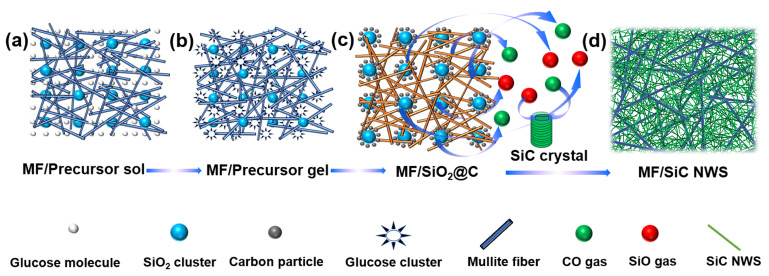
Schematic illustration of the reaction process of SiC NWS: (**a**) MF/Precursor sol, (**b**) MF/Precursor gel, (**c**) MF/SiO_2_@C, (**d**) MF/SiC NWS.

Figure 5 is the schematic illustration of the synthesis mechanism of SiC NWS. As shown in Figure 5a, the SiO_2_ sol and glucose exhibited good water solubility, which was conducive to obtaining a uniformly mixing precursor sol by mechanical stirring. In the drying process (Figure 5b), SiO_2_ clusters were wrapped by glucose clusters owing to the relative excess of glucose and the binding between glucose cluster and SiO_2_ cluster through hydrogen bonding interaction [51]. The close contact between carbon precursor and silicon precursor contributed to the enhancement of reaction activity during the subsequent heat treatment, which provided more chances for synthesizing SiC NWS at relatively low temperatures [52]. The drying was completed, followed by the glucose experiencing a series of pyrolysis and carbonization processes during thermal treatment in Ar [49]. Subsequently, MF/dry gel transformed into MF/SiO_2_@C (Figure 5c). With the increased heat treatment temperature, SiO_2_ particles began to grow and close contact with amorphous carbon, and then the solid–solid reaction started, generating a large amount of reducing gases, such as SiO and CO [47]:

SiO_2_ (s) + C (s) → SiO (g) + CO (g) (1)

The formation of SiC NWS consists of two stages—namely, nucleation and growth. At the nucleation stage, C atoms on the surface of C-coated SiO_2_ nanostructure reacted with SiO gas to form silicon carbide crystal nuclei, providing growth sites for the growth of SiC NWS [21]:

SiO (g) + 2C (s) = SiC (s) + CO (g)(2)

The continuous growth of SiC NWS was through the gas-phase reaction between CO and SiO. In the heat preservation stage, since the sample was prepared in a relatively closed crucible, most of the SiO and CO gases generated by the reaction of SiO_2_@C composites remained in the sample. When the CO gas reached a high degree of saturation, CO and SiO generated a new SiC crystal nucleus, as expressed in Equation (3) [53]. The newly formed crystal nucleus was deposited on the heads of the nanowires to form long SiC NWS.

SiO (g) + 3CO (g) = SiC (s) + 2CO_2_ (g) (3)

The generated CO_2_ using Equation (3) continued to react with the remaining C to produce CO (Equation (4)), which continued to participate in the growth of SiC NWS [48].

CO_2_ (g) + C (s) = 2CO (g) (4)

During the cooling phase, the Gibbs free energy of Equation (5) decreases with the decline in temperature. When the SiO gas was dominant, Equation (5) was favorable [54]. Therefore, a thin layer of amorphous SiO_2_ might be formed on the surface of SiC NWS, following Equation (5), which was consistent with TEM images.

3SiO (g) + CO (g) = SiC (s) + 2SiO_2_(s) (5)

Connecting pores in MF preform facilitated the diffusion of gas products such as SiO, CO, and CO_2_ [55], thereby ensuring that the reaction proceeds smoothly, as well as the uniform distribution of SiC NWS (Figure 5d).

### 3.3. Mechanical Properties of MF/SiC NWS

**Figure 6:**
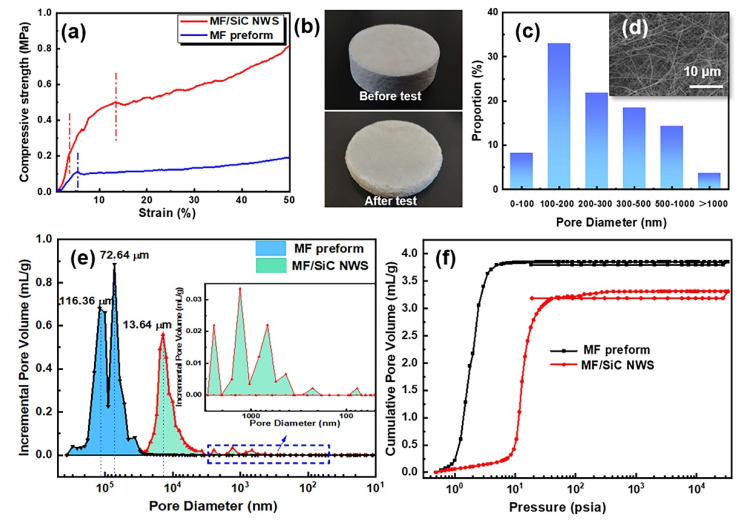
(**a**) Compressive stress–strain curves of MF preform and MF/SiC NWS; (**b**) photographs of the MF/SiC NWS before and after the compressive test; (**c**) pore-size distribution and (**d**) corresponding SEM image of SiC NWS; (**e**) pore-size distributions and (**f**) capillary pressure curve of MF preform and MF/SiC NWS via mercury porosimetry.

The mechanical properties of the thermal insulation material are one of the most vital factors that determine their practical applications. For pure MF preform, high porosity and brittleness lead to relatively low compressive strength. Therefore, as shown in Figure 6a, the compressive stress–strain curve of MF preform only consisted of a linear stage (0–5% strain), and a compaction stage (after 5% strain). By contrast, MF/SiC NWS exhibited higher toughness and ductility, which could effectively counteract the compressive load and enhance the compressive strength. The compression stress–strain curves of MF/SiC NWS included three stages: linear stage (0–3% strain), nonlinear stage (3–15% strain), and compaction stage (after 15% strain). There were two distinct differences between MF/SiC NWS and MF preform in the compression deformation process: (1) in the approximate linear phase, the elastic modulus of MF preform was significantly lower than that of MF/SiC NWS because there was no SiC NWS resisting stress; (2) when the applied strain exceeded 5%, fiber joints of MF preform were destroyed, leading to the failure of the MF preform. However, no destruction was observed (Figure 6b) in MF/SiC NWS even at 60% strain. In summary, SiC NWS were capable of withstanding partial compressive stresses and acted as a buffer against deformation. In addition, MF/SiC NWS exhibited a high compressive strength of 0.47 MPa at 10% strain, which was more than 370% higher than MF preform (0.10 MPa) at room temperature.

The pore-size distribution of MF/SiC NWS was characterized using mercury intrusion porosimeter and SEM image analysis (Figure 6c–e). According to the pore-size distribution curves, MF preform showed a considerable number of macropores with pore sizes of 50–200 μm and two mode values of 72 μm and 116 μm. After in situ synthesis of SiC NWS, it was observed that the macropores in the range of 50–200 μm disappeared in MF/SiC NWS but a noticeable hierarchical pore structure with decreasing size appeared. Specifically, MF/SiC NWS possessed two peak values of 10 μm and 1 μm formed by overlapping mullite fibers and one peak of only 80 nm generated by overlapping mullite fibers and SiC NWS. This was also demonstrated by the results of the capillary pressure curve, as shown in Figure 6f. Hg liquid entered MF preform immediately under very low pressure, and intake increased rapidly, indicating that MF preform possesses a larger pore size and more connected pores than MF/SiC NWS.

### 3.4. Thermal Insulation Performance of MF/SiC NWS

**Figure 7:**
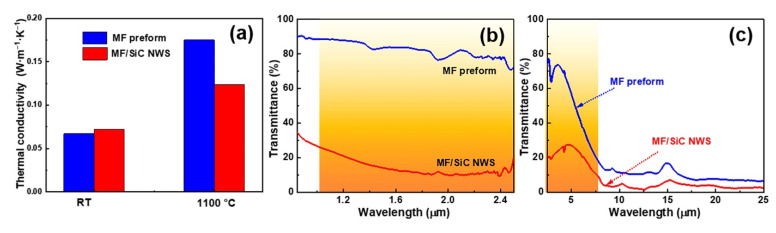
(**a**) The thermal conductivity of MF preform and MF/SiC NWS at room temperature (RT) and 1100 °C; (**b**,**c**) Infrared transmittance spectra of MF preform and MF/SiC NWS.

Thermal conductivities of the MF preform and MF/SiC NWS at room temperature and 1100 °C are given in Figure 7a. The thermal conductivity of MF/SiC NWS was 0.072 W·m^−1^·K^−1^ at room temperature, which was slightly higher than that of MF preform (0.067 W·m^−1^·K^−1^). By contrast, the thermal conductivity of MF/SiC NWS decreased by 0.050 W·m^−1^·K^−1^ (~30%), compared with that of MF preform at 1100 °C. This demonstrated the role of the SiC NWS in improving thermal insulation properties at high temperatures. To verify this, infrared transmittance of MF preform and MF/SiC NWS between 0.85 and 25 μm was measured. In the band of 1–2.5 μm, the results showed that the infrared transmittance of MF/SiC NWS was about 10–20%, and the infrared transmittance of MF preform was about 70–90%, which suggests the infrared transmittance decreased by about 70% after in situ synthesis of SiC NWS (Figure 7b). As shown in Figure 7c, in the band of 2.5–8 μm, the infrared transmittance of MF/SiC NWS changed from 30% to 10% with increasing wavelength, which was lower than that of MF preform (MF preform changed from 80% to 20%). To summarize, the introduction of SiC NWS into the MF preform significantly reduced its infrared transmittance in the 1–8 μm band, indicating MF/SiC NWS exhibited excellent infrared shielding performance in a high-temperature environment.

Generally speaking, the heat transfer of materials mainly consists of solid and gas thermal conduction, thermal radiation, and thermal convection. Thermal convection is negligible in the fiber/nanowire aerogel, because the pores inside the material are micron-sized, further leading that the temperature difference in the pores is very small and almost no airflow can be formed [56]. At room temperature, the heat transfer of materials is mainly accomplished by solid and gas thermal conduction. Solid thermal conduction of fiber/nanowires aerogel is primarily dependent on its intrinsic thermal conductivity, and gas thermal conduction is mainly determined by the bulk density and the pore size. Therefore, at room temperature, there was no improvement in thermal insulation performance after in situ synthesis of SiC NWS for the following two reasons: (1) SiC NWS intrinsic thermal conductivity was relatively high; (2) the bulk density of MF/SiC NWS was 0.22 ± 0.02 g·cm^−3^, which was slightly higher than MF preform (0.20 ± 0.02 g·cm^−3^). However, in a high-temperature environment, the heat transfer of materials is mainly accomplished by thermal radiation. The wavelength of thermal radiation is mainly concentrated at 1–8 μm at high temperatures [31]; in this band, SiC NWS are able to effectively reduce the infrared transmittance, giving rise to the decrease in radiation thermal conduction. Moreover, after the in situ synthesis of SiC NWS, both the decline in pore size and the formation of hierarchical pore structures contributed to the decreasing collision frequency of gas molecules inside the pores of MF/SiC NWS, which is beneficial for restraining the thermal conduction of the gases [12]. Therefore, the high-temperature thermal conductivity of MF/SiC NWS decreased significantly after in situ synthesized SiC NWS.

**Figure 8:**
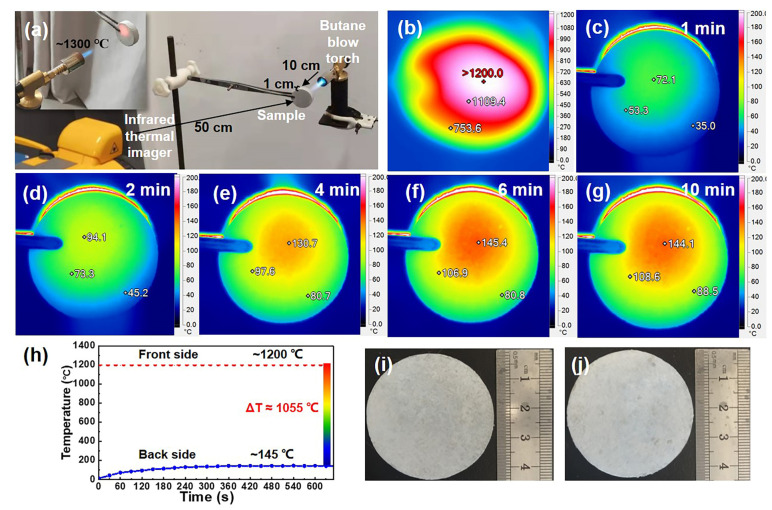
(**a**) Experimental device diagram for recording infrared thermal images of samples; (**b**) infrared thermal image of the front side subjected to the butane blowtorch flame; (**c**–**g**) infrared images of the backside during the 10 min heating process: (**c**) 1 min, (**d**) 2 min, (**e**) 4 min, (**f**) 6 min, and (**g**) 10 min; (**h**) the highest temperature on the backside of the MF/SiC NWS during the heating process; (**i,j**) photographs of the MF/SiC NWS before and after 10 min fire resistance test.

The excellent thermal insulation performance was further confirmed by simulating the high-temperature combustion environment in practical applications. Butane flames were used to simulate extreme high-temperature environments, and an infrared thermal imager was used to record the temperature changes on the back of samples over time. A detailed description of the device is shown in Figure 8a. The flame temperature of the butane spray lamp was about 1300 °C. Under the action of a butane spray lamp flame, the front temperature of MF/SiC NWS with the thickness of 10 mm reached above 1200 °C (Figure 8b). Instead, the maximum temperature on the back of the MF/SiC NWS was only 72 °C (Figure 8c) after heating for 1 min. With the extension of heating time, the maximum temperature on the back of the MF/SiC NWS increased slowly (Figure 8d–f). After 6 min of heating, the maximum temperature on the back of the MF/SiC NWS began to remain unchanged (Figure 8f) and eventually maintained at about 145 °C after 10 min (Figure 8g), which is much lower than the front temperature of 1055 °C (Figure 8h). The sample remained unburned throughout the heating process, and no changes occurred in the shape and size of the samples, except for slight oxidation on the surface of the MF/SiC NWS (Figure 8i,j). The above results suggest that MF/SiC NWS possess good thermal insulation and fire resistance.

### 3.5. High-Temperature Stability of MF/SiC NWS

**Figure 9:**
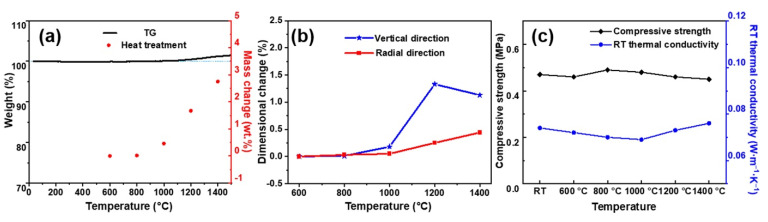
(**a**) TGA curve of the MF/SiC NWS obtained in air atmosphere and mass change after heat treatment for 2 h at different temperatures in air; (**b**) dimension change and (**c**) compressive strength and thermal conductivity of MF/SiC NWS after heat treatment for 2 h at different temperatures in air.

Thermal stability is also an important criterion for evaluating the application safety of thermal protective materials. To demonstrate the thermal stability of MF/SiC NWS, thermogravimetric analysis (TGA) was conducted. As shown in Figure 9a, the weight of the MF/SiC NWS sample achieved almost no changes up to 1100 °C, indicating its excellent thermal stability. From 1100 to 1500 °C, it showed a very small weight change of only 1.4% due to oxidation of the SiC NWS. MF/SiC NWS samples were annealed at 600, 800, 1000 to 1400 °C in the air for 2 h to further study their structural stability. The mass and dimensions change in the samples after being annealed in the air are shown in Figure 9a,b. As the heat treatment temperature increased, the mass and dimensions of the sample increased slightly. After heat treatment at 1400 °C, the mass change rate of the sample was only 2.75%, and the vertical and radial expansion line rates were 1.13% and 0.44%, respectively. These results indicated that MF/SiC NWS had excellent structural stability. The thermal conductivity of MF/SiC NWS was almost invariant after annealing in the air at different temperatures (Figure 9c). The compressive strength of MF/SiC NWS showed a decreasing trend with increasing test temperature, but when the test temperature increased to 1400 °C, the compressive strength of MF/SiC NWS was still up to 0.45 Mpa, with strength retention of 96%, indicating its excellent thermal stability in high temperatures.

## 4. Conclusions

In this study, MF/SiC NWS were prepared by in situ synthesizing SiC NWS in MF preform using a combination of vacuum impregnation and high-temperature pyrolysis. SiC NWS were capable of withstanding compressive stresses, acting as a buffer against deformation. Consequently, MF/SiC NWS exhibited excellent mechanical properties, with the compressive strength of 0.47 MPa at 10% strain, which was over 370% higher than that of MF preform (0.10 MPa). Meanwhile, SiC NWS were uniformly distributed in MF preform, significantly decreasing the pore size and forming hierarchical pore structures, as well as exhibiting a remarkable infrared shielding effect. The infrared transmittance of MF/SiC NWS was reduced by ~70%, compared with MF preform, and MF/SiC NWS exhibited exceptional thermal insulation and thermal stability at 1400 °C. This research provides a new idea for future design and preparation of thermal protection materials suitable for harsh environments.

## Figures and Tables

**Table 1:** Comparison of different types of aerogels along with some of their properties.

Aerogels	Density (g·cm^−3^)	Porosity (%)	Thermal Conductivity at RT (W·m^−1^·K^−^^1^)	Compressive Strength at 10% Strain (MPa)	Upper Limit of Service Temperature (°C)	Refs.
Traditional solid nanoparticle aerogels	0.089–0.18	93.7–95.2%	0.01250–0.0377	0.15–1	600–1000	[5,6,7,9,10,11]
Fiber-reinforced aerogels	0.142–0.52	77.8–88%	0.049–0.236	0.3–1.6	800–1200	[13,15,16,17,18]
Ceramic nanofibrous aerogels	0.0001–0.035	≥99%	0.0157–0.034.6	10^−^^6^–10^−^^3^	1000–1400	[19,20,21,22,23,24,25,26,27]

## Data Availability

Data are contained within the article.
